# Government agencies in the United States are obstructing native species restoration, creating regulatory pits for wildlife

**DOI:** 10.1093/biosci/biaf116

**Published:** 2025-07-16

**Authors:** Jeremy T Bruskotter, L Mark Elbroch, John A Vucetich

**Affiliations:** School of Environment and Natural Resources, Ohio State University, Columbus, Ohio, United States; Puma Program director, Panthera, New York, New York, United States; College of Forest Resources and Environmental Science, Michigan Technological University, Houghton, Michigan, United States

Rapidly changing societal and environmental conditions over the past few centuries have severely degraded ecosystems and accelerated losses of biodiversity (Bogoni et al. 2020). The resulting biodiversity crisis encapsulates both global extinction risk, which is estimated to be orders of magnitude above the background rate (Pimm et al. 2014), as well as range losses due to local extirpations (Vucetich et al. 2023). The importance of the latter may be under appreciated. For example, one study found most native mammalian fauna, including species of low conservation concern, were extirpated from more than 50% of the coterminous United States (Ceballos and Ehrlich 2002). Consequently, conservation strategies to repair damages to native ecosystems in the United States must include active restoration of species.

Since its inception, the primary federal policy instrument in the United States for restoring native species has been the Endangered Species Act of 1973 (ESA). However, ongoing policy developments are eroding the nation's capacity to protect and restore species. Over the past two decades, the agencies charged with implementing the ESA (i.e., the US Fish and Wildlife Service and the National Marine Fisheries Service; hereafter, USFWS and NMFS) have promulgated rules that would limit their obligations for species restoration, in part by reinterpreting the legal definition of an endangered species so as to narrow its application and preclude the need for restoration in most instances (Vucetich et al. 2023). Now, the Trump administration seeks to further limit application of the ESA both through executive orders and by changing long-standing administrative rules (e.g., redefining *harm* under the ESA in order to make it easier to modify species’ habitat).

Efforts to restrict application of the ESA are not limited to the Executive Branch. To wit: Congress has long failed to provide the USFWS and NMFS with sufficient funds to administer the ESA (Eberhard et al. 2022). Moreover, Congress is currently considering numerous bills that would further restrict the ESA, such as the ESA Amendments Act of 2025 (H.R. 1897), which would repeal automatic protections for threatened species and hand over their management to states. These efforts have occurred even as research shows that four in five Americans support the ESA, and this number has not wavered for three decades (Vucetich et al.^ 2025^).

Collectively, these actions amount to an abdication of federal leadership with regards to imperiled species conservation. In the absence of federal leadership, the obligation to conserve and restore native fauna falls to the states. However, state governments appear reluctant to adequately invest in species restoration—even for species that could be readily restored (e.g., habitat generalists). Consider these recent examples:

In February of 2024, the Pennsylvania Game Commission tabled a plan to reintroduce the American marten (*Martes americana*) despite the fact that more than 90% of nearly 1000 public comments were supportive. The Game Commission cited a survey of hunters reporting that only 37% supported marten restoration, whereas 32% opposed it (Danahy 2024).

In the mid-1990s, the USFWS considered reintroducing gray wolves (*Canis lupus*) to the state of Colorado. In doing so, researchers surveyed residents in 1994 and found that more than 60% of the respondents supported restoration (Pate et al. 1996). From that time forward, the Colorado Wildlife Commission stymied reintroduction efforts, citing concerns about livestock and big game interests (Blevins 2019). It took a 2020 ballot measure to force the state to reintroduce wolves. Moreover, when the state of Colorado reached out to nearby states to acquire wolves for reintroduction, those states declined to provide wolves in support of Colorado's reintroduction effort despite state management plans aggressively seeking to minimize wolf populations.

Beyond their reluctance to restore species, many states manage species in a manner that appears designed to inhibit them from reoccupying historic range—even where habitat is suitable. For example, the objective of Wyoming's management of gray wolves includes a stable population in only approximately 20% of the state, and unlimited killing in the remainder of the state outside national parks and tribal lands to “minimize wolf conflicts with livestock and maintain wild ungulate herds” (Mills and Atkinson 2023).

Consider also how states manage the easternmost populations of cougar (*Puma concolor*). South Dakota limits cougar harvest in the Black Hills, which composes less than 10% of the state along its western border, and allows unlimited, year-round hunting in the remainder of the state (South Dakota Game, Fish, and Parks 2019). Nebraska approved a cougar hunt in 2024 of their easternmost population, which is estimated to be just 27 animals, including kittens (Alliance Times-Herald 2024). Oklahoma recently passed legislation (S.B. 1073) that allows lottery hunting of cougars despite the fact that they have yet to even establish a breeding population.

In the language of ecology, a *predator pit* occurs when predators prevent prey from reaching a carrying capacity determined by available resources (i.e., type 3 functional response; Holling 1965). We contend that, by analogy, wildlife management agencies are creating regulatory pits for wildlife (i.e., management regimes that effectively lower animal densities and inhibit reoccupation of historic range) and thereby, the restoration of biodiversity. These actions beg a simple question: Why? Such management is often justified in terms of maintaining “socially acceptable” wildlife populations and “minimizing conflicts” with people, leaving the impression that state wildlife priorities reflect the will of their citizens. However, states may have far more mandate to restore native biodiversity than they realized, as was indicated by a recent study that asked the residents of nine US states to prioritize a list of wildlife management activities. That study showed that the highest priority among the respondents was the restoration of locally eradicated or imperiled species (Carlson et al. 2023).

We contend that state wildlife agencies’ failure to restore native species stems, in part, from their prioritization of so called game (i.e., species that can be trapped or hunted). One piece of evidence for this view is state funding priorities. The National Wildlife Federation's “State of Wildlife” reports that, on average, state wildlife agencies spend approximately 10% of their funds on the conservation of nongame wildlife (inclusive of law enforcement to protect these species), whereas the bulk of funding goes to hunted game species (Edelson 2025). Moreover, many states spend substantial funds to introduce nonnative animals (e.g., ring-necked pheasant, *Phasianus colchicus*) solely for the benefit of human hunters. Pennsylvania alone spent more than $4 million on stocking pheasants in FY2023, operating the program at a loss (Bresswein 2024). In another case, the Utah Division of Wildlife Resources introduced nonnative mountain goats to state lands sitting adjacent to the Manti-La Sal National Forest over the US Forest Service's protests that the goats would negatively affect native species (Nie et al. 2020). Mountain goats, of course, are prized by sport hunters. Indeed, the Utah Division of Wildlife Resources touts demand for permits and high hunter success rates in its explanation for why goats were introduced to the state (Utah Division of Wildlife Resources 2018). These cases serve to illustrate where state wildlife agencies’ priorities tend to lie.

Human-caused habitat loss and overexploitation are major causes of the biodiversity crisis, and mitigating the biodiversity crisis will require restoration on a scale never before seen. Fortunately, many species of wildlife in need of restoration are ecological generalists for which habitat exists and the threat of overexploitation has subsided or can be sufficiently mitigated—at least in some contexts. Frustratingly, in many cases, the restoration and recovery of native wildlife in the United States are being obstructed by the very agencies charged with wildlife conservation. Specifically, the systematic neglect of the ESA, coupled with the Trump administration's current efforts to undermine it, is shifting the burden to states steeped in a culture that prioritizes the production of game.

The more general problem is one the conservation community has wrestled with since its inception: how to fund the conservation of species that lack obvious economic value. By tying conservation funding to sport hunting through excise taxes on firearms, the United States has virtually guaranteed game favoritism—a situation McCabe (1931) predicted (and decried) even before the Pittman–Roberston Act was passed in 1937.

Revitalizing wildlife conservation in the United States will require policy changes at both the federal and state levels. Federal actions include abandoning efforts to weaken the ESA, including the USFWS and NMFS's weak interpretation of the legal definition of an endangered species; adequately funding and fully implementing the ESA; and passing federal legislation that provides funding for states to restore native species. State-level actions include reforming state wildlife agencies to explicitly prioritize the mitigation of the biodiversity crisis over provisioning game for recreation; reducing hunting pressures on native species that are reoccupying lost range (e.g., puma), thereby allowing for natural recolonization; and developing governance processes that are inclusive and do not advantage particular interests (e.g., hunters, trappers; Bruskotter et al. 2022). Unfortunately, such changes seem unlikely until the general public and rank-and-file politicians view the biodiversity crisis to be as concerning as the climate crisis.

## Data Availability

No new data were generated or analysed in support of this viewpoint.

## References

[bib1] Alliance Times-Herald . Commissioners Approve 2025 Mountain Lion Season. Alliance Times-Herald (26 June 2024). https://alliancetimes.com/commissioners-approve-2025-mountain-lion-season.

[bib2] Blevins J. 2019. Colorado wildlife officials are reluctant to OK gray wolf reintroduction. So advocates want voters to do it. Colorado Sun (25 April 2019). https://coloradosun.com/2019/04/25/colorado-gray-wolf-reintroduction-ballot-proposal.

[bib3] Bogoni JA, Peres CA, Ferraz KM. 2020. Effects of mammal defaunation on natural ecosystem services and human well being throughout the entire neotropical realm. Ecosystem Services 45: 101173.

[bib4] Bresswein K. WATCH: Pennsylvania pheasant stocking keeps alive hunting tradition rooted in the Lehigh Valley. Lehighvalleylivecom (20 October 2024). www.lehighvalleylive.com/outdoors/2024/10/watch-pennsylvania-pheasant-stocking-keeps-alive-hunting-tradition-rooted-in-the-lehigh-valley.html.

[bib5] Bruskotter JT, Nelson MP, Peterson MN, Peterson TR, Serfass TL, Sullivan L, Vucetich JA. 2022. Beyond Game Management: Toward a More Inclusive Ethic for Wildlife Conservation. School of Environment and Natural Resources, Ohio State University. Report no. 09.03.2022.

[bib6] Carlson SC, Vucetich JA, Elbroch LM, Perry S, Roe LA, Butler T, Bruskotter JT. 2023. The role of governance in rewilding the United States to stem the biodiversity crisis. BioScience 73: 879–884. 38162572 10.1093/biosci/biad099 PMC10755707

[bib7] Ceballos G, Ehrlich PR. 2002. Mammal population losses and the extinction crisis. Science 296: 904–907. 11988573 10.1126/science.1069349

[bib8] Danahy A. 2024. PA Game Commission tables plans to reintroduce the American Marten. Allegheny Front (22 February 2024). www.alleghenyfront.org/pa-game-commission-reintroduction-american-marten.

[bib9] Eberhard EK, Wilcove DS, Dobson AP. 2022. Too few, too late: US Endangered Species Act undermined by inaction and inadequate funding. PLOS ONE 17: e0275322. 36223374 10.1371/journal.pone.0275322 PMC9555635

[bib10] Edelson N. 2025. Toolkit for wildlife conservation leaders: Working together to reverse America's Wildlife crisis and strengthen state fish and wildlife agencies for the next 100 years. National Wildlife Federation (15 May 2025). https://statewildlifetoolkit.nwf.org.

[bib11] Holling CS. 1965. The functional response of predators to prey density and its role in mimicry and population regulation. Memoirs of the Entomological Society of Canada 45: 3–60.

[bib12] McCabe TT. 1931. More Game Birds in America, Inc. Condor 33: 259–261.

[bib13] Mills KJ, Atkinson CD. 2023. Wyoming Gray Wolf Monitoring and Management 2023 Annual Report. Wyoming Game and Fish Department.

[bib14] Nie M, Pitt K, Barns C, Haber J, Joly J, Zellmer S. 2020. Response to Kisonak's “Fish and Wildlife in Management on Federal Lands: The authorities and responsibilities of State Fish and Wildlife agencies”. Environmental Law 50: 973–997.

[bib15] Pate J, Manfredo MJ, Bright AD, Tischbein G. 1996. Coloradans’ attitudes toward reintroducing the gray wolf into Colorado. Wildlife Society Bulletin 24: 421–428.

[bib16] Pimm SL, Jenkins CN, Abell R, Brooks TM, Gittleman JL, Joppa LN, Raven PH, Roberts CM, Sexton JO. 2014. The biodiversity of species and their rates of extinction, distribution, and protection. Science 344:1246752. 24876501 10.1126/science.1246752

[bib17] South Dakota Game , Fish, and Parks. 2019. South Dakota Mountain Lion Management Plan, 2019–2029. South Dakota Game, Fish, and Parks. Report no. 2019-06.

[bib18] Utah Division of Wildlife Resources . 2018. Utah Mountain Goat Statewide Management Plan. Utah Division of Wildlife Resources.

[bib19] Vucetich JA, Bruskotter JT, Arrivo N, Phillips M. 2023. A proposed policy for interpreting “significant portion of its range” for the U.S. Endangered Species Act, 1973. Georgetown Environmental Law Review 36: 85–128.

[bib20] Vucetich JA, Bruskotter JT, Wilson RS, Elbroch LM, Feltz A, Offer-Westort T. 2025. Support for the U.S. Endangered Species Act is high and steady over the past three decades. Conservation Letters 18: e13111.

